# Conformational Preference of Lithium Polysulfide Clusters Li_2_S_*x*_ (*x* = 4–8) in Lithium–Sulfur Batteries

**DOI:** 10.1021/acs.inorgchem.3c04537

**Published:** 2024-02-28

**Authors:** Xinru Peng, Jiayao Li, Jingshuang Dang, Shiwei Yin, Hengyan Zheng, Changwei Wang, Yirong Mo

**Affiliations:** † Key Laboratory for Macromolecular Science of Shaanxi Province, School of Chemistry & Chemical Engineering, Shaanxi Normal University, Xi’an 710119, China; ‡ Department of Nanoscience, Joint School of Nanoscience and Nanoengineering, University of North Carolina at Greensboro, Greensboro, North Carolina 27401, United States

## Abstract

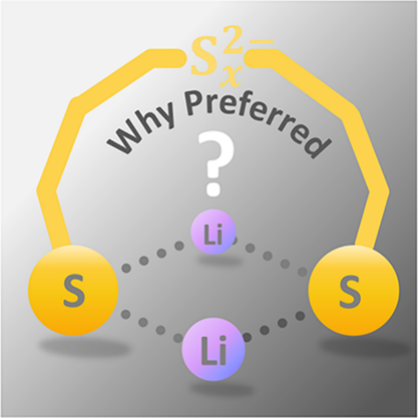

Structures are of fundamental importance for diverse studies of lithium polysulfide clusters, which govern the performance of lithium–sulfur batteries. The ring-like geometries were regarded as the most stable structures, but their physical origin remains elusive. In this work, we systematically explored the minimal structures of Li_2_S_*x*_ (*x* = 4–8) clusters to uncover the driving force for their conformational preferences. All low-lying isomers were generated by performing global searches using the ABCluster program, and the ionic nature of the Li···S interactions was evidenced with the energy decomposition analysis based on the block-localized wave function (BLW-ED) approach and further confirmed with the quantum theory of atoms in molecule (QTAIM). By analysis of the contributions of various energy components to the relative stability with the references of the lowest-lying isomers, the controlling factor for isomer preferences was found to be the polarization interaction. Notably, although the electrostatic interaction dominates the binding energies, it contributes favorably to the relative stabilities of most isomers. The Li^+^···Li^+^ distance is identified as the key geometrical parameter that correlates with the strength of the polarization of the S_*x*_^2–^ fragment imposed by the Li^+^ cations. Further BLW-ED analyses reveal that the cooperativity of the Li^+^ cations primarily determines the relative strength of the polarization.

## Abstract 2

Li_2_S_*x*_ (*x* = 4−8) clusters, which govern the performance of lithium−sulfur batteries, were computationally studied with an energy decomposition approach to uncover the driving force for their conformational preferences. It was found that the controlling factor for isomer preferences is the polarization of the S_*x*_^2−^ fragment imposed by the Li^+^ cations, whose strength is correlated with the Li^+^···Li^+^ distance.

## 1 Introduction

Lithium–sulfur batteries^1−6^ have attracted a great deal of attention because of their remarkable theoretical capacity, energy density, and low cost.^7−12^ Still, the development of lithium–sulfur batteries faces a series of challenges on the path toward realization, and it is thus imperative to understand the reaction mechanisms therein.^2,13−16^ In particular, the lithium polysulfide clusters,^1,17−19^ which are produced in the charge/discharge processes, dramatically influence the performance of batteries.^14,20^ Significant efforts have been dedicated to comprehending their structures,^21,22^ spectra,^23−27^ properties, related interactions,^21,28−30^ and redox mechanisms.^31,32^ In all of these studies, the geometries of lithium polysulfide clusters, especially the lowest-lying structures, are of fundamental importance.

**Scheme 1:**
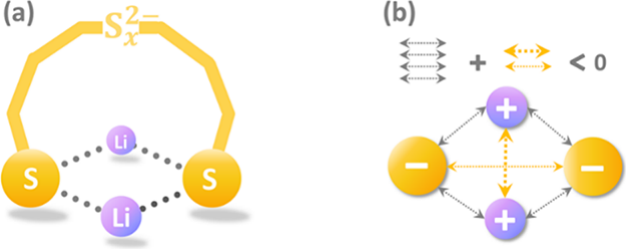
(a) Ring-like Structure of Li_2_S_*x*_ (*x* = 4–8) and (b) Electrostatic Compression Model

Studies have shown that the most stable isomers of Li_2_S_*x*_ (*x* = 4–8) possess ring-like geometries (Scheme 1a), in which each Li^+^ is bound to both terminal sulfur atoms of the S_*x*_^2–^ chain and keeps a limited distance from the other Li^+^.^25,28,31,33^ Seemingly, this ring-like configuration aligns with the concept of electrostatic compression,^34^ which states that the overall electrostatic attraction can be enhanced when ions are crosswise-arranged, since the intercation and interanion repulsions can be overcome by the cation–anion attractions. Therefore, the importance of electrostatic interaction in stabilizing the lowest-lying isomers can be deduced because of the quadrilateral binding pattern (Scheme 1b), which possesses crosswise-stacked ions and subsequently maximizes the electrostatic stability. However, a thorough explanation for the isomer preference requests a comprehensive comparison among all possible low-lying isomers, with all physical factors affecting the stability inspected. Moreover, as a ring structure is composed of three components including two Li^+^ and one dianion S_*x*_^2–^, the cooperative effect in three-body interactions^35^ must be examined.

However, the conformational space of Li_2_S_*x*_ (*x* = 4–8) clusters becomes intricate with *x* increasing due to the wide range of likely geometries exhibited by the S_*x*_^2–^ dianions^36−39^ along with their multiple bonding sites for lithium cations. Thus, identifying low-lying isomers is a vital prerequisite for the study of conformational preferences. It is conventional to build up stable isomers based on chemical knowledge^23,24^ or generate various random structures for further optimizations and screening.^27,32^ Nevertheless, arbitrariness and incompleteness are inevitable for the structures constructed manually, and it is nearly impossible to achieve ergodic sampling on any potential energy surface due to the vast number of local minima for large clusters. In this regard, global search algorithms are invaluable, as they can efficiently navigate energy landscapes, offering us the most stable geometries and other low-lying isomers. For instance, the most stable structures of the (Li_2_S)_*n*_ (*n* = 1–10) complexes were successfully determined by employing CALYPSO software,^40^ which is usually used for the predictions of crystal structures but valid for clusters as well.^41−45^ In addition, global optimizations have been utilized for searching crystalline structures of lithium polysulfides.^26,46,47^ The importance of global optimization has stimulated the development of dedicated software,^48−52^ among which the ABCluster is an ideal choice for chemical clusters.^53,54^ ABCluster employs a novel swarm intelligence algorithm^55^ and supports molecular mechanics and semiempirical and quantum mechanics calculations, allowing it to handle arbitrarily complicated components and topologies. Furthermore, the software is designed in a black-box manner to ensure user-friendliness.

We note that previous studies have analyzed some specific isomers of Li_2_S_*x*_ by examining the geometrical parameters, energies, and electron populations.^21,22,29,31,32^ Clearly, a more insightful understanding of the conformational preferences can be obtained by incorporating bonding analysis methods^56−68^ into a comprehensive study of all low-lying isomers. In this regard, the ab initio valence bond (VB) theory stands out as an ideal option. It offers a chemically intuitive solution for a molecular wave function, which is a linear combination of Lewis structures constructed from strictly localized and fully optimized orbitals.^69,70^ As the simplest ab initio VB method, the block-localized wave function (BLW) retains the localized orbitals but simplifies the multireference wave function to a single determinant, making it computationally efficient.^71−74^ Its associated energy decomposition (BLW-ED in short) approach can decompose the binding energy into physically meaningful energy components^75−77^ and has provided a multitude of new insights into the conventional and unconventional interactions.^78−82^ Moreover, the BLW method has the capabilities of geometrical optimization and spectral calculation for the strictly electron-localized state.

In this work, we explored the conformational preferences of Li_2_S_*x*_ (*x* = 4–8) clusters by comparing each of the low-lying isomers with the most stable structure in the same cluster. To achieve this, global optimizations were performed to identify all low-lying structures, and the BLW-ED approach was subsequently employed to elucidate the underlying physical factors that dominate the Li···S interactions and, critically, the relative stability. Furthermore, the cooperative effect in the accumulated Li···S interactions and its contribution to the conformational preferences were elucidated with the BLW-ED approach. Structures of low-lying isomers were thoroughly examined to reveal the key geometrical parameter(s) that can rationalize the primary factor for the conformational preferences.

## 2 Methods and Computational Details

### 2.1 BLW-ED Approach

The binding energy (Δ*E*_b_) represents the overall energy variation upon the formation of a complex from fully relaxed monomers and can be decomposed into five terms as expressed in eq 1. The deformation energy (Δ*E*_def_) in eq 1 is usually defined as the energy cost to deform the optimal monomers to the geometries in the complex. For each *x*, the most stable isomer of the S_*x*_^2–^ dianion was selected as the universal reference to evaluate the deformation energy, ensuring that the binding energy can serve as an exact measure of the relative stability among isomers. Except for the deformation energy, the rest of the binding energy is defined as the interaction energy (Δ*E*_int_), which can be further decomposed. In detail, the frozen energy (Δ*E*_F_) is the energy change resulting from putting distorted monomers together without disturbing all orbitals. This frozen component comprises electrostatic interaction (Δ*E*_ele_), Pauli exchange repulsion (Δ*E*_Pauli_), and most electron correlation (Δ*E*_corr_) at the DFT level (eq 2) and can be further decomposed utilizing the XEDA program.^67^ Polarization energy (Δ*E*_pol_) refers to the stabilization arising from the relaxation of electron densities within individual monomers, induced by the electric field exerted by the others. The complex can be further stabilized when electron delocalization among monomers is allowed. This part of energy lowering is defined as the charge transfer energy (Δ*E*_CT_), to which the basis set superposition error (BSSE) correction is fully assigned. Finally, the difference in Grimme’s dispersion corrections between the complex and distorted monomers is denoted as the dispersion correction component (Δ*E*_D3_). The combination of electron correlation (Δ*E*_corr_) and dispersion correction component (Δ*E*_D3_) corresponds to the total electron correlation energy (Δ*E*_ec_) in accordance with their physical interpretations. In summary, the detailed BLW-ED scheme is expressed in eq 3.
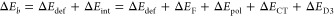
1
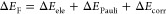
2
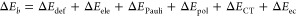
3

Since each Li_2_S_*x*_ cluster consists of three monomers, there is a three-body cooperativity to be analyzed. The cooperative component in each energy term, denoted with the superscript “C”, can be evaluated by excluding all pairwise contributions (Δ*E*_y_^ij^) from the total value (Δ*E*_*y*_) defined in eq 3 (y = int, ele, Pauli, pol, CT, and ec) as
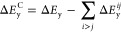
4

The cooperative component in the interaction energy can be taken as the energy measure of the overall cooperative effect. A negative value of the cooperative component in the interaction energy indicates the positive cooperativity that reinforces the stability of system, while a positive value suggests the presence of anticooperativity.

### 2.2 Computational Details

Global searches for the isomers of S_*x*_^2–^ and Li_2_S_*x*_ (*x* = 4–8) clusters were performed using ABCluster combined with Gaussian16 at the M06-2X/def2-SVP theoretical level^83,84^ with Grimme’s D3 dispersion correction incorporated.^85^ One hundred generations were accomplished in the global searches. For each cluster, the lowest-lying structure and its isomers, which lie within 50.00 kcal/mol higher than the most stable one, were retained for further optimizations at the M06-2X-D3/def2-TZVP level using GAMESS(US).^86^ Vibrational frequencies were computed to ensure that optimal isomers were true minima on potential energy surfaces. Localized bond force constants were derived from the local vibrational mode theory.^59,68^ In addition, we reoptimized the low-lying isomers of Li_2_S_4_ at the M06-2X-D3/def2-TZVPD level of theory and found that their relative stabilities were barely changed by the additional diffuse functions (Figure S1). Relative Gibbs free energies and binding energies were calculated by taking the values of the lowest-lying isomer as references. There are excellent linear correlations between relative Gibbs free energies and binding energies, with the slopes close to one (Figure S2). Thus, we focused on analyzing the binding energies to explain the isomer preference.

BLW calculations were carried out using the in-house version of GAMESS(US), with three blocks defined for monomers (one S_*x*_^2–^ and two Li^+^). For the lowest-lying isomers, the geometries of the strictly electron-localized states (i.e., no electron movement between S_*x*_^2–^ and Li^+^ cations) with the BLW method were optimized and compared with the regular DFT results, with the electron movement from S_*x*_^2–^ to Li^+^ cations allowed through the minimized root-mean-square value of deviations (RMSD) of atom coordinates. This was accomplished with the visual molecular dynamics (VMD) program.^87^ QTAIM theory was employed to inspect the nature of the Li···S interactions using Multiwfn software.^88,89^

## 3 Results and Discussion

**Figure 1:**
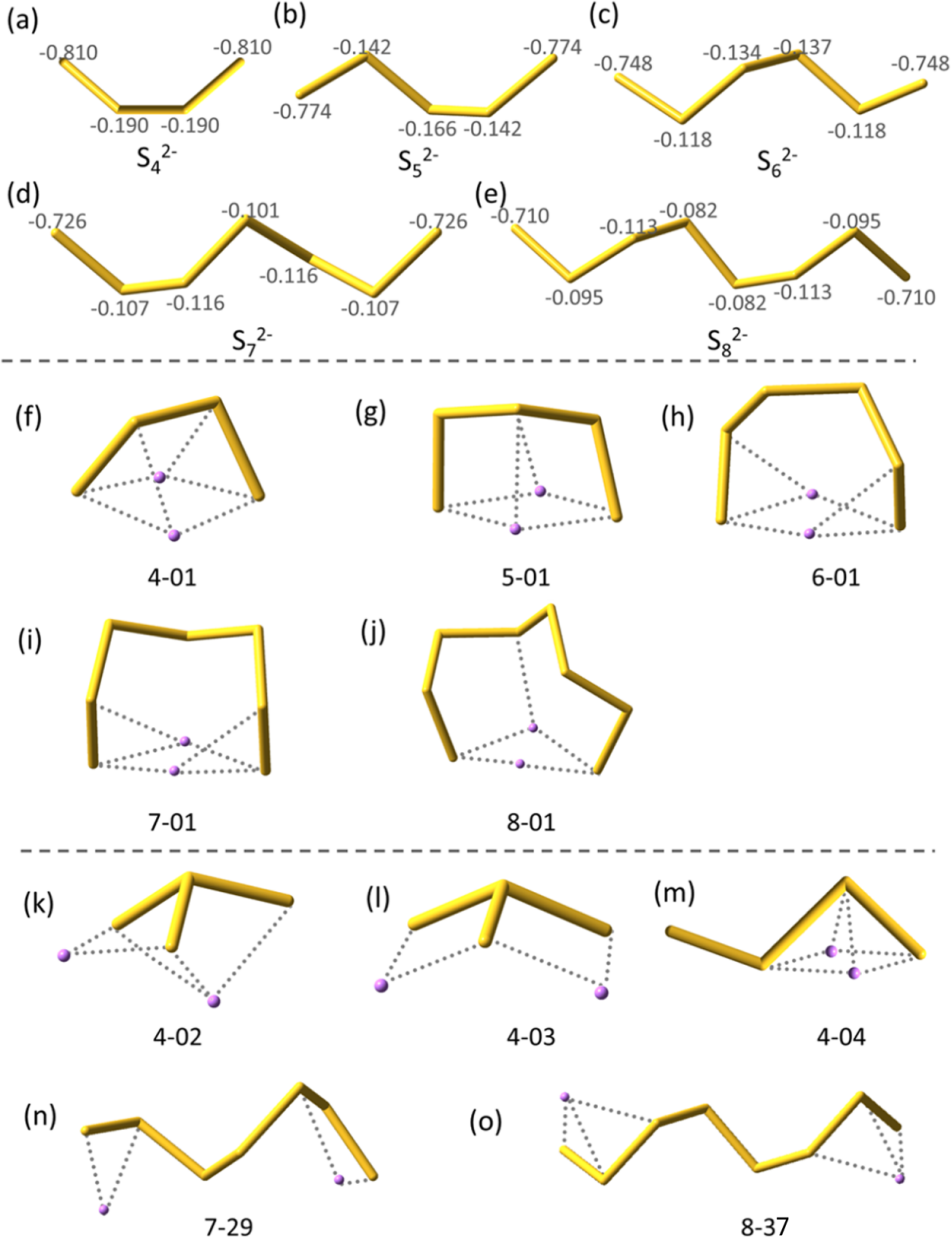
Optimized geometries of (a–e) global minima of S_*x*_^2–^ dianions (*x* = 4–8) with charges derived from the natural population analysis, (f–j) global minima of Li_2_S_*x*_ (*x* = 4–8) clusters, (k–m) additional low-lying isomers of Li_2_S_4_, and (n, o) chain-like structures of Li_2_S_7_ and Li_2_S_8_.

Selected geometries are listed in Figure 1. For clarity, yellow tubes are used for the S_*x*_^2–^ framework, while Li···S interactions are denoted with dotted lines when any Li···S distance is shorter than the sum of their ionic radii (2.60 Å). The low-lying isomers of a Li_2_S_*x*_ (*x* = 4–8) cluster are denoted as “*x* – *n*”, where *x* represents the number of sulfur atoms and *n* is the sequential number arranged in an ascending order of relative energies. Geometries of all low-lying isomers are shown in Figure S3.

As shown in Figure 1a–e, chain structures of S_*x*_^2–^ were proved to be the lowest-lying conformers through global optimizations. This is reasonable because the net negative charges are mainly localized on the terminal sulfur atoms in chain structures, implying minimized Coulombic repulsion. The most stable structures of Li_2_S_*x*_ (*x* = 4–8) clusters are shown in Figure 1f–j, which exhibit the ring-like characteristics (Scheme 1a). The geometries of low-lying isomers become increasingly diverse with the cluster size increasing (Figure S3), as the S_*x*_^2–^ framework can adopt either various nonbranching structures or branching structures. Notably, the quadrilateral binding pattern (Scheme 1b) was found in most isomers, while geometries with far-separated Li^+^ cations were also observed. Here, the simplest cluster Li_2_S_4_ can serve as an illuminating example. Isomers 4-01 and 4-04 are built up with the nonbranching conformers of S_4_^2–^ and closely located Li^+^ cations, while a trigonal pyramidal (branching) dianion is observed in isomers 4-02 and 4-03, where Li^+^ cations are sufficiently separated. In the chain-like structures of Li_2_S_7_ and Li_2_S_8_ (Figure 1n,o), two Li^+^ cations are separated further away. Although these two isomers take the most favorable chain conformations for the S_*x*_^2–^ fragment, they exhibit much lower stabilities (34.9 and 38.1 kcal/mol) compared with their corresponding lowest-lying structures.

**Figure 2:**
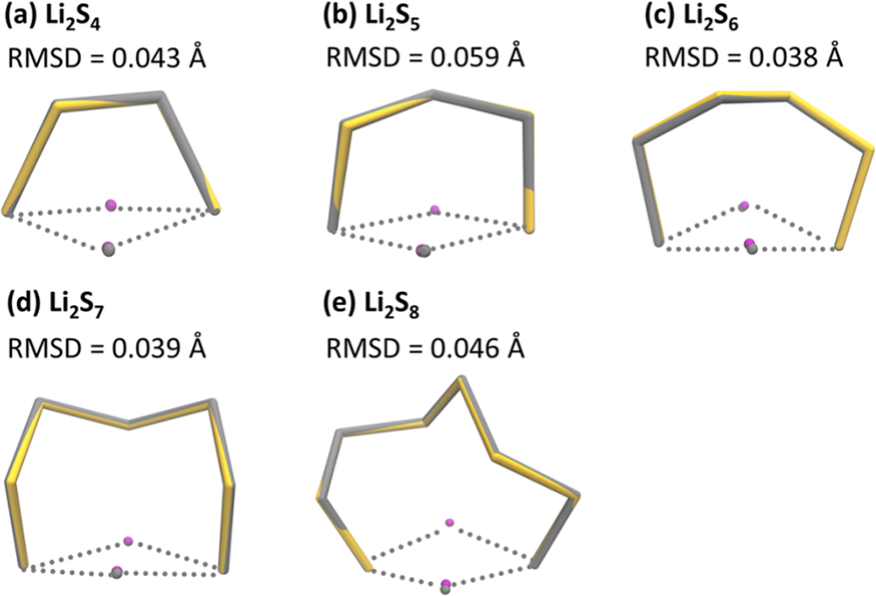
Comparison of optimal structures of electron-delocalized (in yellow) and electron-localized states (in gray) for the lowest-lying (a) Li_2_S_4_, (b) Li_2_S_5_, (c) Li_2_S_6_, (d) Li_2_S_7_, and (e) Li_2_S_8_, with the corresponding RMSD values denoted.

Bonding analyses were conducted on the lowest-lying isomers to explore the nature of the Li···S interactions therein. Individual Li···S bonds (Figure 1f–j) in the quadrilateral bonding regions were confirmed through the bond force constants and the bond critical points (BCPs), which exhibit positive Laplace values (Table 1), implying closed-shell interactions. Remarkable strengths of the accumulated Li···S interactions were evidenced by the extremely high binding energies ranging from −370.9 to −414.2 kcal/mol. Obviously, the binding energies are predominantly governed by electrostatic interactions, suggesting the ionic nature of Li···S bonds. Polarization and charge transfer interactions emerge as the second- and third-most important attractive forces, with electron correlation contributing the least to the overall stability. The governing role of electrostatic interaction in the binding energy was observed across all low-lying isomers (Table S1). For each of the lowest-lying isomers, the optimal structures of both electron-delocalized and electron-localized states were compared, resulting in minimized RMSD of atomic coordinates (less than 0.059 Å; Figure 2). Hence, covalency contributes negligibly to the Li···S bonds in the most stable isomers, where the Li···S bonds are ionic.

**Table 1:** BLW-ED Results (in kcal/mol), Average Bond Force Constants (*k*, in mDyn/ Å), Average Values of Electron Density (ρ, in a.u.), and Laplace Values (∇^2^ρ, in a.u.) at the BCPs of All Li···S Interactions in the Lowest-Lying Isomers

*x*	Δ*E*_def_	Δ*E*_ele_	Δ*E*_Pauli_	Δ*E*_pol_	Δ*E*_CT_	Δ*E*_ec_	Δ*E*_b_	*k*	ρ	∇^2^ρ
4	8.7	–377.1	55.9	–80.8	–15.1	–5.8	–414.2	0.498	0.025	0.123
5	15.3	–360.6	55.3	–88.9	–14.3	–5.6	–398.8	0.502	0.025	0.118
6	18.4	–341.5	52.3	–96.7	–15.6	–5.6	–388.7	0.485	0.025	0.120
7	25.6	–331.0	53.1	–104.9	–15.0	–5.7	–377.9	0.415	0.025	0.112
8	27.9	–328.3	55.5	–105.8	–14.6	–5.6	–370.9	0.483	0.025	0.114

Electrostatic interaction is also primarily responsible for the reduction of the binding energy in the lowest-lying isomer with the size of the cluster growing (Table 1). In fact, there is a good linear correlation between Δ*E*_ele_ and Δ*E*_b_. This trend is understandable because the net negative charges on the terminal sulfur atoms decrease with increasing size of the S_*x*_^2–^ dianion (Figure S4a). In sharp contrast, as the size of the cluster grows, polarization is significantly enhanced due to the increased average polarizability of S_*x*_^2–^ (Figure S4b). In fact, an excellent linear correlation with a negative slope between Δ*E*_pol_ and Δ*E*_b_ can be observed. Another energy term that varies with *x* is the deformation energy. It increases as the dianion S_*x*_^2–^ gets longer, implying enhanced strain from Li_2_S_4_ to Li_2_S_8_. This is reasonable because a longer S_*x*_^2–^ dianion requires more deformation cost to transform from the most favorable chain-like structure to the semicircular geometry in the complex Li_2_S_*x*_. The rest of the three energy components, including the charge transfer, Pauli exchange repulsion, and electron correlation, are contingent on orbital overlaps between blocks and barely vary by the size of the S_*x*_^2–^ dianion.

**Figure 3:**
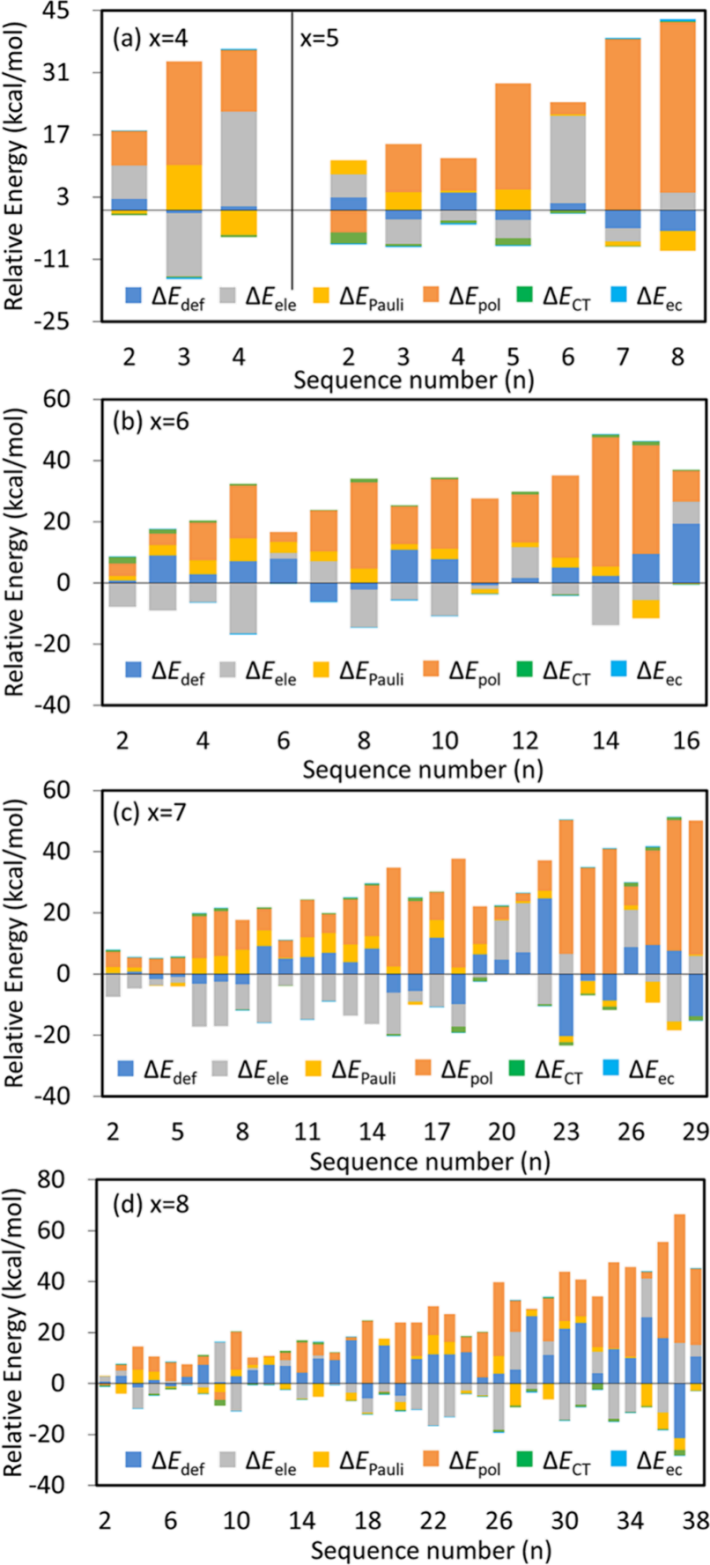
Relative values of all energy components in the BLW-ED analyses for (a) Li_2_X_4_ and Li_2_X_5_, (b) Li_2_X_6_, (c) Li_2_X_7_, and (d) Li_2_X_8_.

Figure 3 displays the relative values of individual energy components with the most stable isomers of Li_2_S_*x*_ clusters as the respective references. Therefore, positive values mean unfavorable impact on the conformational preference. A total of 95 low-lying isomers were retained in this work of five Li_2_S_*x*_ clusters (*x* = 4–8), and 90 relative binding energies were analyzed as five lowest-lying isomers serve as zero points. Polarization emerges as the primary determinant for conformational preferences in most cases (58/90), followed by the deformation energy, which dominates the relative binding energies of 23 isomers. Usually, relative stabilities of isomers with branched S_*x*_^2–^ frameworks are dominated by the deformation energy, because the semicircular geometries (unbranching) of the S_*x*_^2–^ fragment in the lowest-lying structures undergo less deformation and thus are much more stable than the branching structures. Interestingly, the electrostatic interaction, which rules the Li···S interactions, governs the relative stabilities of only 9 isomers and contributes favorably to the relative stabilities of 63 isomers. In other words, most geometries exhibit stronger electrostatic interactions than the quadrilateral pattern of the Li···S bonds (Scheme 1b) in all lowest-lying isomers. For instance, isomer 4-03 (Figure 1l) features much stretched distance between Li^+^ cations compared to the lowest-lying structure (3.824 Å vs 2.841 Å) but with more attractive electrostatic interaction (−391.2 vs −377.1 kcal/mol). Notably, the global minima exhibit the strongest polarization among all low-lying isomers when *x* equals 4, 6, and 7. For Li_2_S_5_ and Li_2_S_8_, polarization interaction in the most stable isomers consistently ranks among the top three in all low-lying structures. None of the relative binding energies are governed by the Pauli exchange repulsion, charge transfer interaction, or electron correlation.

**Figure 4:**
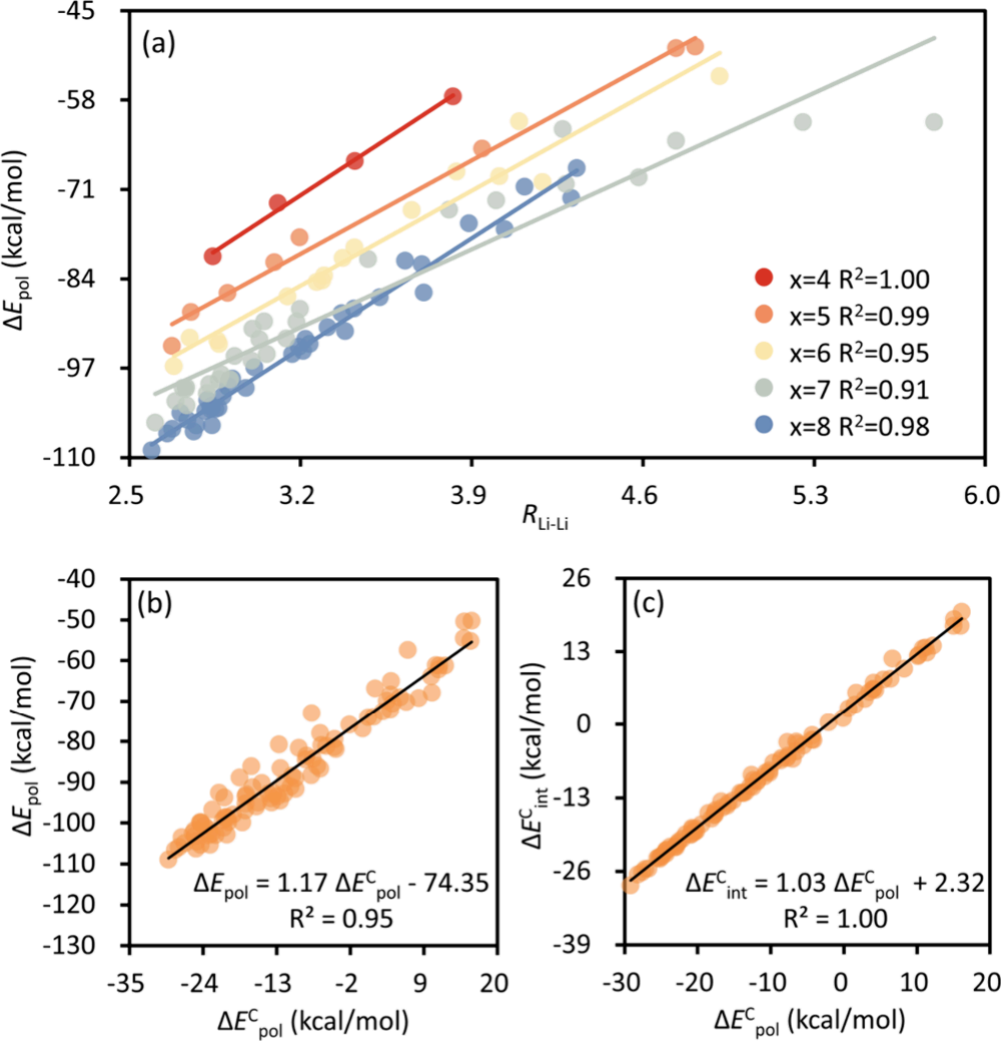
(a) Correlation between the polarization energy and the Li···Li distance for all low-lying isomers of each Li_2_S_*x*_ (*x* = 4–8) cluster. (b) Correlation between the polarization energy and its cooperative component and (c) correlation between cooperative components of total interaction and polarization energies of all isomers.

To understand the geometric correlations with the polarization energy term, we explored various structural parameters and eventually identified the Li^+^···Li^+^ distance as the principal index for polarization. Figure 4a shows that for each cluster, the strengths of polarization correlate with the Li^+^···Li^+^ distances approximately in linear manners. The interpretation for this correlation is that a close arrangement of the two cations leads to an enhanced resultant electric field, which ultimately exerts an enhanced force for the polarization within the S_*x*_^2–^ dianion. This also explains the remarkable polarization energies observed in the lowest-lying isomers aligned with the characteristic quadrilateral binding pattern (Scheme 1b), where Li^+^ cations are closely positioned. The strength of polarization can be quantitatively elucidated by examining the cooperative effect. Figure 4b shows that the polarization energy beautifully correlates with its cooperative component linearly. This suggests that the relative strength of polarization is primarily governed by the cooperative effect. Furthermore, the cooperative components of both total interaction and polarization energies also exhibit a linear correlation (Figure 4c), indicating that the overall cooperative effect is predominantly dominated by polarization interactions. We further assessed the cooperative components of all of the physical factors in the BLW-ED analyses (Table S2). The frozen energy exhibits a trivial many-body effect (−1.22 to 0.33 kcal/mol) due to the use of unadjusted orbitals in its evaluation. Consequently, we did not further decompose the frozen term in the analysis of the cooperative effect. The dispersion correction, which has minimal impact on the interaction energy, also contributes little to the cooperative effect (0.00–0.07 kcal/mol). Differently, charge transfer exhibits an anticooperative effect (0.51–5.97 kcal/mol) in all isomers due to the competition between the Li^+^ cations in taking electrons from the S_*x*_^2–^ dianion.

**Figure 5:**
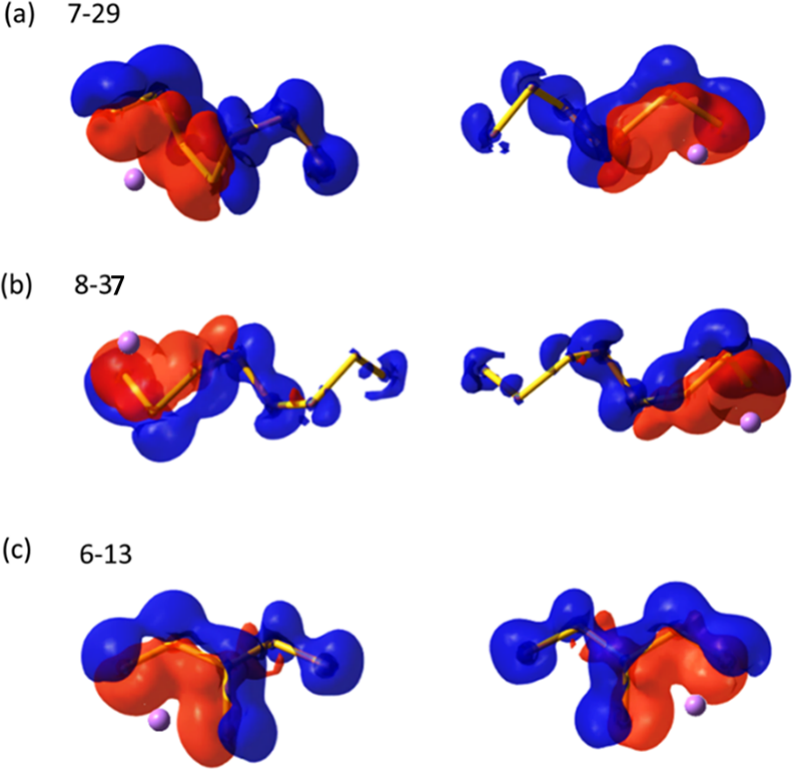
Electron density difference (EDD) maps representing electron density variations (polarizations) within the S_*x*_^2–^ fragment induced by an individual Li^+^ cation for isomers (a) 7-29, (b) 8-37, and (c) 6-13 (the red color means a gain of electron density, while the blue color represents a loss of electron density with the isovalue of 0.001 e Å^–3^ selected).

The anticooperative effect was observed in the interaction energies of 23 isomers, marked with “ACE” in Figure S3. In these cases, Li^+^ cations are usually distanced from each other, with the S_*x*_^2–^ dianion (or portion of it) sandwiched in between. Chain-like isomers and isomer 6-13 serve as illustrative examples, as depicted in Figure 5, where the electron density variation or polarization within the S_*x*_^2–^ dianion caused by individual Li^+^ cations is represented. Clearly, the Li^+^ cations pull the electron density of the S_*x*_^2–^ dianion in nearly opposite directions, indicating the presence of anticooperative effect. It is necessary to emphasize that all lowest-lying isomers exhibit a considerable cooperative effect due to the characteristic quadrilateral binding pattern where two Li^+^ cations are closely arranged and polarize the S_*x*_^2–^ dianion in the same direction, while the anticooperative cases are comparatively much less stable as two Li^+^ polarize the S_*x*_^2–^ dianion in different directions.

## 4 Conclusions

Low-lying isomers of lithium polysulfide clusters Li_2_S_*x*_ (*x* = 4–8) were thoroughly searched, and the characteristic ring-like geometries with a quadrilateral binding pattern were observed in all lowest-lying isomers. The Li···S interactions in the most stable isomers were studied and found to be ionic. Electrostatic interaction also rules the weakening of Li···S interactions as the size of cluster grows, as the negative charges on the terminal sulfur atoms of the S_*x*_^2–^ fragment decrease with *x* increasing.

Key factors for the conformational preferences were explored by examining the contributions of individual energy components to the relative stabilities with references to the most stable isomers. Polarization stabilization governs the relative stabilities of most low-lying isomers (58/90), with the deformation energy playing the second-most important role. Pauli exchange repulsion, charge transfer, and total electron correlation barely contribute to the relative stabilities. Notably, the electrostatic interaction, which dominates the binding energies of all low-lying isomers, contributes favorably to the relative stability in most cases (65/90). The Li···Li distance is a crucial geometrical parameter determining the strength of the polarization interaction, which rules the conformational preferences. A close arrangement of two Li^+^ results in an enhanced resultant electric field, which polarizes the dianion S_*x*_^2–^. This cooperativity effect between two cations was quantitatively verified, and the strength of the polarization was primarily determined by its cooperative component, which exhibited a linear correlation with the polarization energy. We also investigated the cases with the anticooperative effect, which results from the long separation of two Li^+^ cations, which polarize the dianion S_*x*_^2–^ in nearly opposite directions. In summary, the conformational preference of Li_2_S_*x*_ (*x* = 4–8) clusters mainly originates from the cooperative effect of the polarization interaction, which is strengthened by the closely located Li^+^ cations within the quadrilateral binding region of the ring-like geometries.

## Supporting Information Available

Relative binding energies of Li_2_S_4_ with various basis sets and the M06-2X-D3 functional, correlations between the relative values of Gibbs free energies and binding energies for all low-lying isomers of Li_2_S_*x*_ and their geometries (*x* = 4–8), natural charges of S and polarizabilities of the S_*x*_^2–^ fragment in the lowest-lying isomers of Li_2_S_*x*_, BLW-ED results of all low-lying isomers, and Cartesian coordinates of optimal structures studied in this work (PDF)
