# Supplementary material for: Conformational Preference of Lithium Polysulfide Clusters Li2Sx (x = 4–8) in Lithium–Sulfur Batteries
Source: Inorg Chem. 2024 Feb 28;63(10):4716–24. doi: 10.1021/acs.inorgchem.3c04537 (PMC10934799; doi:10.1021/acs.inorgchem.3c04537)
Supplement: Supplementary file 1 — ic3c04537_si_001.pdf [file ic3c04537_si_001.pdf]

Supporting Information for:

Conformational Preference of Lithium Polysulfide Clusters Li_2S_x (x=4-8) in Lithium-Sulfur Batteries

Xinru Peng[a], Jiayao Li[a], Jingshuang Dang[a], Shiwei Yin[a], Hengyan Zheng[a], Changwei Wang*[a] and Yirong Mo*[b]

[a] Key Laboratory for Macromolecular Science of Shaanxi Province, School of Chemistry & Chemical Engineering, Shaanxi Normal University, Xi'an 710119, China.

[b] Department of Nanoscience, Joint School of Nanoscience & Nanoengineering, University of North Carolina at Greensboro, Greensboro, NC 27401, USA.

AUTHOR INFORMATION

Corresponding Author

* snnu.changweiwang@snnu.edu.cn; y_mo3@uncg.edu.

Contents

Figure S1. Relative binding energies (in kcal/mol) of the Li_2S_4, calculated by using the M06-2X-D3 functional and different basis sets.---S-3

Figure S2. Correlations between the relative values of Gibbs free energies and binding energies for all low-lying isomers of (**a**) Li_2S_4, (**b**) Li_2S_5, (**c**) Li_2S_6, (**d**) Li_2S_7, and (**e**) Li_2S_8.---S-3

Figure S3. Geometries of all low-lying Li_2S_x (x=4-8) isomers. Different background were colors used for isomers with different governing factors for their relative stabilities (orange for polarization, blue for deformation grey for electrostatic interaction). Yellow background color was utilized for the lowest-lying isomers (references). The relative binding energies were denoted in the parentheses, and "ACE" was marked on cases with anti-cooperative effect.---S-4~S-6

Figure S4. Variations in (**a**) the average charge of terminal sulfur atoms in the deformed S_x^{2-} fragment from the natural population analysis and (**b**) the average polarizabilities of the S_x^{2-} dianions in the lowest-lying isomers of Li_2S_x, with increasing cluster size (x).---------------S-7

Table S1. BLW-ED results (in kcal/mol) of all low-lying isomers.---------------------S-8~S-10

Table S2. The cooperative components of energy terms in the BLW-ED scheme (in kcal/mol).-- ----------S-11~S-13

Table S3. The xyz coordinates (in Å) of the lowest-lying isomers of S_x^{2-} and Li_2S_x (x=4-8) clusters.---S-14~S-15

Additional Figures

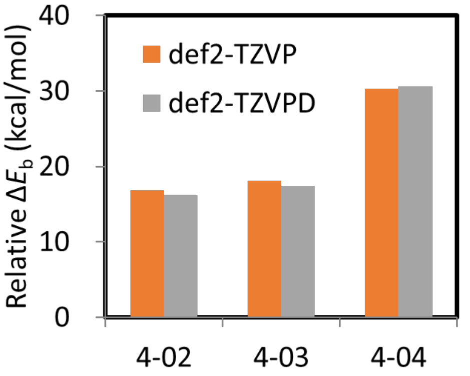

Figure S1. Relative binding energies (in kcal/mol) of the Li_2S_4, calculated by using the M06-2X-D3 functional and different basis sets.

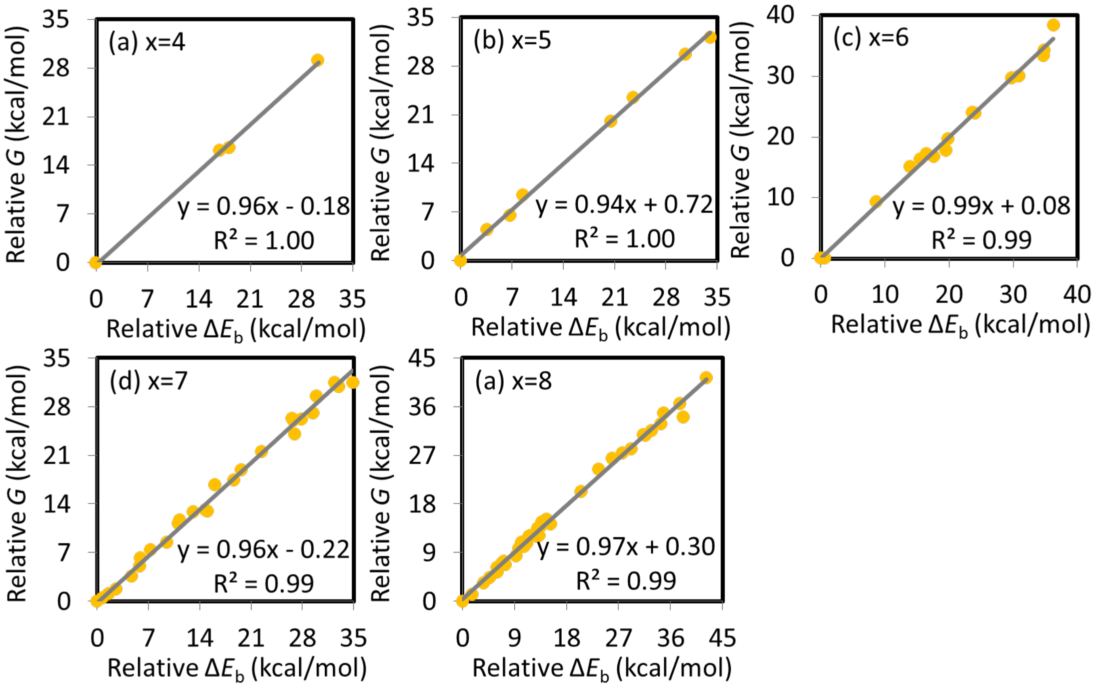

Figure S2. Correlations between the relative values of Gibbs free energies and binding energies for all low-lying isomers of (**a**) Li_2S_4, (**b**) Li_2S_5, (**c**) Li_2S_6, (**d**) Li_2S_7, and (**e**) Li_2S_8.

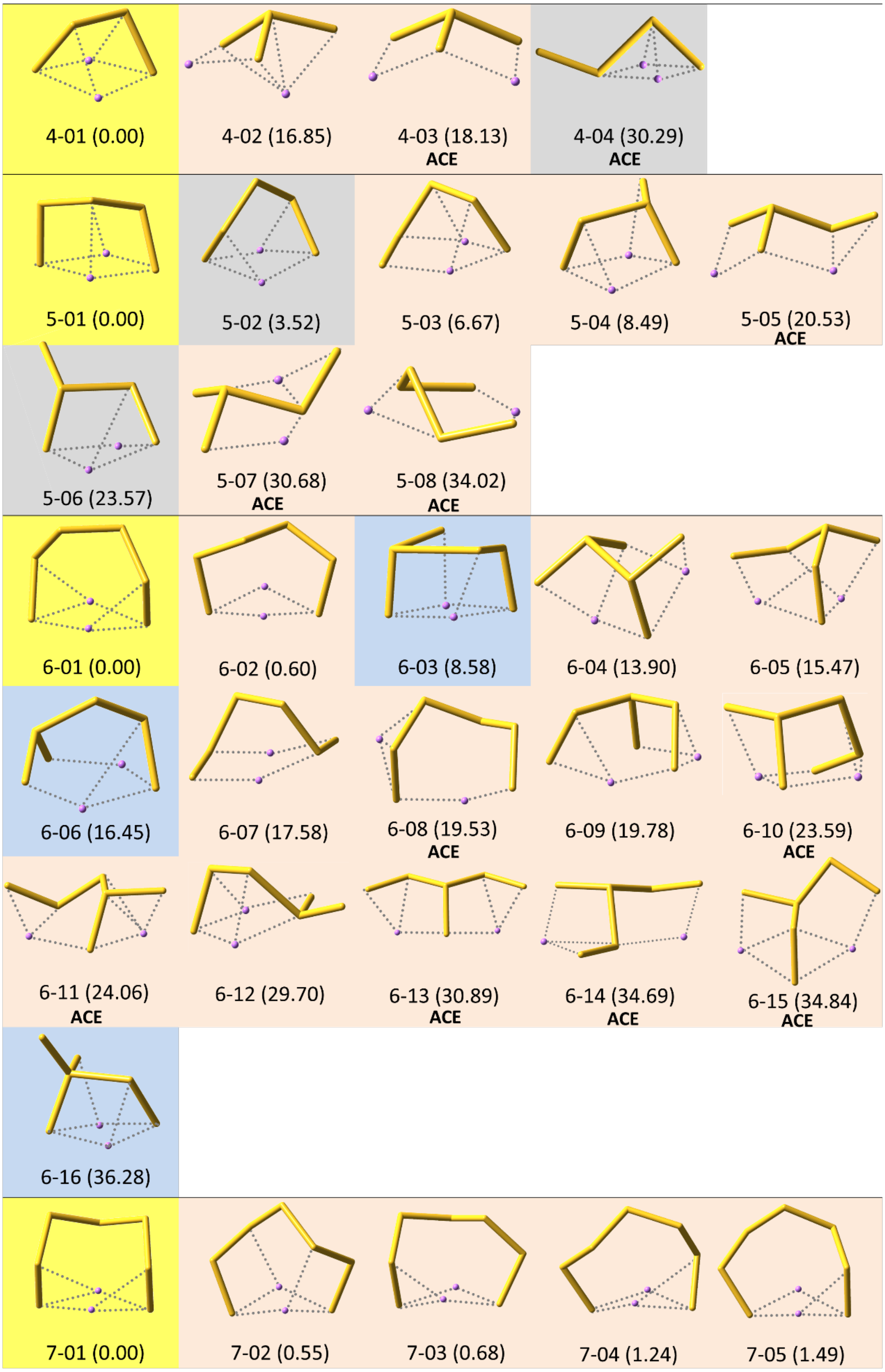
4-01 (0.00)
4-02 (16.85)
4-03 (18.13)
ACE
4-04 (30.29)
ACE
5-01 (0.00)
5-02 (3.52)
5-03 (6.67)
5-04 (8.49)
5-05 (20.53)
ACE
5-06 (23.57)
5-07 (30.68)
ACE
5-08 (34.02)
ACE
6-01 (0.00)
6-02 (0.60)
6-03 (8.58)
6-04 (13.90)
6-05 (15.47)
6-06 (16.45)
6-07 (17.58)
6-08 (19.53)
ACE
6-09 (19.78)
6-10 (23.59)
ACE
6-11 (24.06)
ACE
6-12 (29.70)
6-13 (30.89)
ACE
6-14 (34.69)
ACE
6-15 (34.84)
ACE
6-16 (36.28)
7-01 (0.00)
7-02 (0.55)
7-03 (0.68)
7-04 (1.24)
7-05 (1.49)

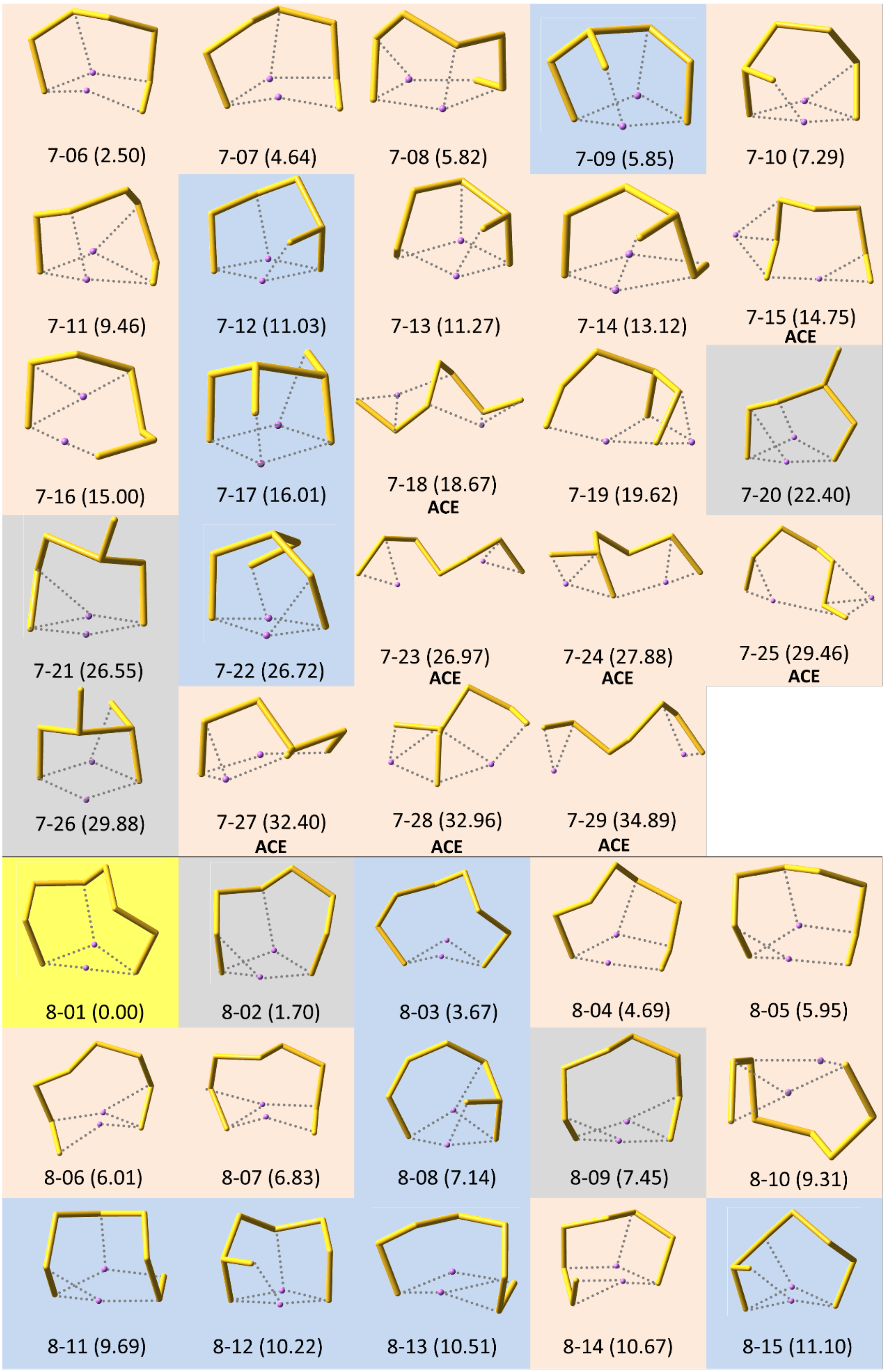
7-06 (2.50)
7-07 (4.64)
7-08 (5.82)
7-09 (5.85)
7-10 (7.29)
7-11 (9.46)
7-12 (11.03)
7-13 (11.27)
7-14 (13.12)
7-15 (14.75)
ACE
7-16 (15.00)
7-17 (16.01)
7-18 (18.67)
ACE
7-19 (19.62)
7-20 (22.40)
7-21 (26.55)
7-22 (26.72)
7-23 (26.97)
ACE
7-24 (27.88)
ACE
7-25 (29.46)
ACE
7-26 (29.88)
7-27 (32.40)
ACE
7-28 (32.96)
ACE
7-29 (34.89)
ACE
8-01 (0.00)
8-02 (1.70)
8-03 (3.67)
8-04 (4.69)
8-05 (5.95)
8-06 (6.01)
8-07 (6.83)
8-08 (7.14)
8-09 (7.45)
8-10 (9.31)
8-11 (9.69)
8-12 (10.22)
8-13 (10.51)
8-14 (10.67)
8-15 (11.10)

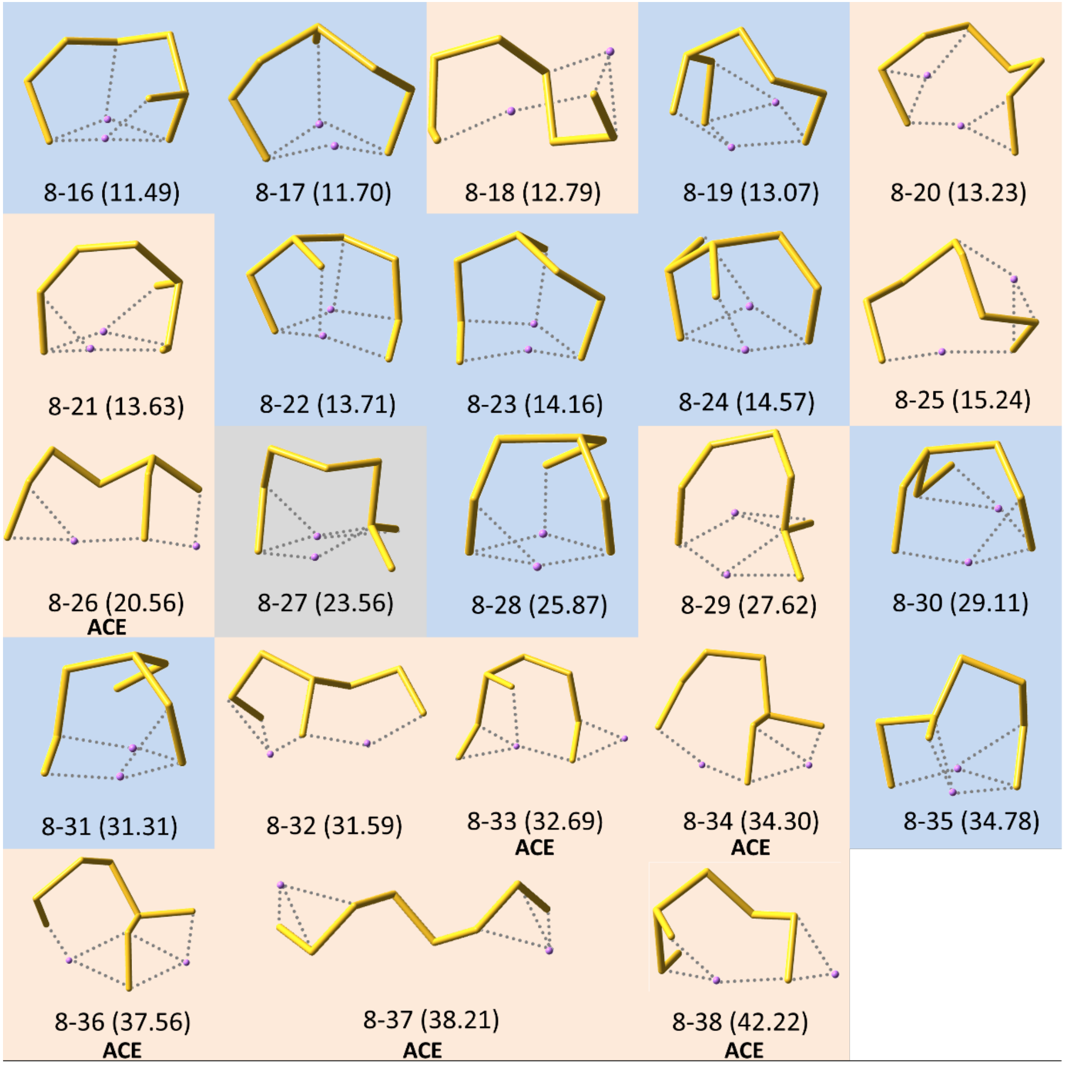

Figure S3. Geometries of all low-lying Li_2S_x (x=4-8) isomers. Different background were colors used for isomers with different governing factors for their relative stabilities (orange for polarization, blue for deformation grey for electrostatic interaction). Yellow background color was utilized for the lowest-lying isomers (references). The relative binding energies were denoted in the parentheses, and "ACE" was marked on cases with anti-cooperative effect.

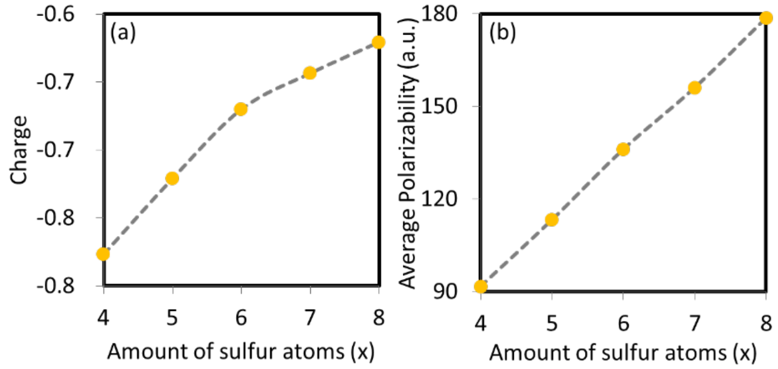

Figure S4. Variations in (**a**) the average charge of terminal sulfur atoms in the deformed S_x^{2-} fragment from the natural population analysis and (**b**) the average polarizabilities of the S_x^{2-} dianions in the lowest-lying isomers of Li_2S_x, with increasing cluster size (x).

Additional Tables

Table S1. BLW-ED results (in kcal/mol) of all low-lying isomers.

x-n	ΔE_{def}	ΔE_{ele}	ΔE_{Pauli}	ΔE_{pol}	ΔE_{CT}	ΔE_{ec}	ΔE_{b}
x=4							
4-01	8.66	-377.08	55.94	-80.76	-15.08	-5.79	-414.12
4-02	11.24	-369.60	55.25	-72.99	-15.42	-5.75	-397.27
4-03	8.00	-391.23	66.16	-57.45	-15.35	-6.13	-396.00
4-04	9.63	-355.78	50.40	-66.89	-15.58	-5.62	-383.84
x=5							
5-01	15.30	-360.62	55.26	-88.85	-14.30	-5.62	-398.86
5-02	18.17	-355.43	58.43	-93.78	-16.83	-5.87	-395.34
5-03	13.22	-366.07	59.31	-77.94	-14.86	-5.83	-392.19
5-04	19.30	-362.84	55.67	-81.58	-15.00	-5.89	-390.37
5-05	13.12	-364.74	59.96	-65.05	-15.78	-5.84	-378.35
5-06	16.87	-340.91	55.56	-86.08	-14.95	-5.76	-375.30
5-07	11.28	-363.62	54.29	-50.24	-14.39	-5.47	-368.18
5-08	10.64	-356.63	50.87	-50.46	-14.11	-5.13	-364.85
x=6							
6-01	18.37	-341.54	52.32	-96.72	-15.62	-5.57	-388.81
6-02	19.08	-349.37	53.88	-92.55	-13.70	-5.51	-388.21
6-03	27.40	-350.53	55.63	-92.96	-14.16	-5.57	-380.24
6-04	21.23	-347.84	56.80	-84.25	-15.07	-5.73	-374.91
6-05	25.37	-358.10	59.85	-79.41	-15.15	-5.86	-373.36
6-06	26.24	-339.66	55.93	-93.46	-15.64	-5.72	-372.36
6-07	12.33	-334.51	55.68	-83.50	-15.45	-5.73	-371.23
6-08	16.23	-353.79	56.96	-68.42	-14.50	-5.72	-369.29
6-09	29.14	-346.96	54.32	-84.45	-15.27	-5.81	-369.03
6-10	26.13	-352.22	55.66	-74.00	-15.02	-5.73	-365.23
6-11	17.63	-342.75	50.79	-69.07	-15.70	-5.60	-364.76
6-12	20.04	-331.54	53.83	-80.96	-14.81	-5.61	-359.11
6-13	23.37	-345.18	55.59	-69.91	-16.01	-5.73	-357.93
6-14	20.70	-355.38	55.43	-54.50	-14.82	-5.50	-354.12
6-15	27.80	-347.17	46.35	-61.11	-14.44	-5.35	-353.98
6-16	37.70	-334.37	51.83	-86.59	-15.33	-5.72	-352.53
x=7							
7-01	25.58	-331.04	53.09	-104.91	-15.01	-5.64	-378.01
7-02	25.76	-338.47	55.24	-99.97	-14.49	-5.44	-377.45
7-03	26.38	-335.79	54.49	-101.81	-14.98	-5.53	-377.32
7-04	23.97	-333.04	52.77	-99.88	-14.99	-5.51	-376.77
7-05	24.63	-332.91	51.82	-99.74	-14.63	-5.60	-376.52
7-06	22.37	-345.08	58.27	-91.26	-14.14	-5.57	-375.50
7-07	23.14	-345.55	58.91	-90.19	-14.06	-5.52	-373.36
7-08	22.31	-339.32	61.07	-95.25	-15.24	-5.67	-372.19

7-09	34.64	-346.88	58.31	-97.91	-14.52	-5.72	-372.16
7-10	30.55	-334.74	53.53	-99.30	-15.15	-5.53	-370.72
7-11	31.08	-345.84	59.68	-92.81	-14.88	-5.70	-368.55
7-12	32.49	-339.84	59.53	-98.70	-14.72	-5.65	-366.98
7-13	29.40	-344.59	58.98	-90.24	-14.58	-5.63	-366.74
7-14	33.89	-347.39	57.19	-88.38	-14.51	-5.60	-364.89
7-15	19.58	-344.61	55.59	-72.60	-15.45	-5.69	-363.26
7-16	19.99	-334.46	52.17	-81.11	-14.00	-5.51	-363.00
7-17	37.49	-341.79	58.89	-95.93	-14.76	-5.82	-362.00
7-18	15.82	-338.46	55.17	-69.25	-16.84	-5.70	-359.34
7-19	32.01	-332.23	56.49	-92.68	-16.11	-5.79	-358.39
7-20	30.21	-318.11	53.32	-100.69	-14.66	-5.60	-355.61
7-21	32.61	-315.00	53.86	-102.40	-14.89	-5.56	-351.46
7-22	50.26	-340.87	55.57	-94.93	-15.48	-5.76	-351.29
7-23	5.27	-324.43	51.05	-61.20	-16.08	-5.57	-351.03
7-24	23.46	-331.21	48.90	-70.22	-15.44	-5.53	-350.12
7-25	16.98	-331.10	51.10	-63.93	-16.01	-5.51	-348.55
7-26	34.35	-318.82	54.49	-98.63	-13.91	-5.53	-348.13
7-27	34.99	-333.59	46.32	-73.92	-13.99	-5.33	-345.61
7-28	33.19	-346.58	50.22	-62.19	-14.09	-5.50	-345.04
7-29	11.81	-325.15	53.60	-61.19	-16.40	-5.71	-343.12
x=8							
8-01	27.93	-328.32	55.52	-105.84	-14.64	-5.53	-371.01
8-02	28.71	-326.39	55.67	-106.51	-15.12	-5.56	-369.32
8-03	30.94	-326.35	51.58	-103.48	-14.48	-5.43	-367.33
8-04	26.38	-336.51	61.08	-96.91	-14.65	-5.60	-366.32
8-05	29.21	-332.33	58.79	-99.71	-15.32	-5.58	-365.06
8-06	26.90	-327.39	54.79	-98.51	-15.15	-5.52	-365.00
8-07	30.59	-328.76	55.54	-101.05	-14.77	-5.60	-364.18
8-08	35.33	-329.86	53.05	-102.60	-14.12	-5.56	-363.87
8-09	28.46	-312.75	52.10	-108.95	-16.90	-5.51	-363.56
8-10	30.82	-339.09	58.04	-91.03	-14.62	-5.71	-361.70
8-11	33.15	-327.48	56.69	-102.78	-15.17	-5.60	-361.31
8-12	35.32	-328.60	58.48	-105.30	-14.92	-5.65	-360.79
8-13	34.80	-326.01	53.20	-102.94	-13.81	-5.63	-360.50
8-14	32.16	-334.07	54.95	-93.90	-13.81	-5.55	-360.34
8-15	37.71	-327.03	50.36	-101.65	-13.72	-5.46	-359.91
8-16	37.06	-328.87	55.54	-102.93	-14.63	-5.57	-359.52
8-17	44.87	-331.80	52.43	-104.55	-14.71	-5.44	-359.31
8-18	22.11	-333.79	54.90	-81.38	-14.40	-5.58	-358.26
8-19	42.83	-331.40	58.11	-106.22	-15.40	-5.75	-357.95
8-20	23.20	-330.90	52.50	-81.89	-15.00	-5.57	-357.78
8-21	37.58	-338.48	56.63	-92.75	-14.65	-5.60	-357.38
8-22	39.31	-344.81	63.19	-94.52	-14.65	-5.72	-357.30

8-23	39.35	-341.27	60.53	-94.95	-14.65	-5.75	-356.85
8-24	40.20	-330.97	54.25	-99.87	-14.34	-5.60	-356.44
8-25	30.41	-333.01	55.24	-88.32	-14.32	-5.64	-355.77
8-26	31.71	-346.47	62.51	-76.78	-15.41	-5.89	-350.45
8-27	33.50	-313.63	46.74	-93.46	-15.07	-5.41	-347.45
8-28	54.39	-330.36	57.88	-105.29	-15.79	-5.86	-345.14
8-29	39.05	-322.92	49.29	-89.00	-14.29	-5.40	-343.39
8-30	49.42	-342.59	58.67	-86.69	-14.89	-5.71	-341.90
8-31	51.69	-336.64	58.22	-91.59	-15.47	-5.79	-339.70
8-32	31.95	-319.93	57.41	-85.98	-16.94	-5.80	-339.42
8-33	41.30	-342.20	56.04	-72.22	-15.53	-5.60	-338.32
8-34	37.84	-339.52	56.13	-70.63	-14.77	-5.65	-336.71
8-35	53.88	-313.06	46.59	-103.21	-14.98	-5.34	-336.23
8-36	45.61	-339.61	48.86	-67.90	-14.74	-5.57	-333.46
8-37	6.54	-312.41	50.67	-55.24	-16.69	-5.63	-332.87
8-38	38.43	-323.72	52.82	-75.90	-14.86	-5.45	-328.79

Table S2. The cooperative components of energy terms in the BLW-ED scheme (in kcal/mol).

x-n	$\Delta E^{C}{}_{F}$	$\Delta E^{C}{}_{pol}$	$\Delta E^{C}{}_{CT}$	$\Delta E^{C}{}_{D3}$	$\Delta E^{C}{}_{int}$
x=4					
4-01	-0.40	-12.61	4.04	0.00	-8.97
4-02	-0.37	-7.73	4.97	0.00	-3.13
4-03	-0.85	6.64	5.97	0.00	11.77
4-04	-1.22	1.72	5.23	0.00	5.73
x=5					
5-01	-0.11	-18.53	2.92	0.00	-15.72
5-02	0.16	-20.80	2.30	0.00	-18.34
5-03	-0.32	-6.49	3.71	0.00	-3.10
5-04	-0.33	-9.64	3.14	0.00	-6.83
5-05	-0.50	4.10	3.88	0.00	7.49
5-06	-0.47	-16.78	3.62	0.00	-13.64
5-07	-0.56	16.15	4.47	0.02	20.07
5-08	-0.75	15.07	4.44	0.02	18.78
x=6					
6-01	-0.06	-22.58	1.90	0.00	-20.74
6-02	-0.11	-21.62	2.27	0.00	-19.46
6-03	0.02	-17.54	2.35	0.00	-15.17
6-04	-0.26	-7.60	2.32	0.00	-5.54
6-05	-0.27	-4.33	2.68	0.00	-1.91
6-06	0.10	-17.64	1.94	0.00	-15.61
6-07	0.33	-8.51	2.24	0.00	-5.95
6-08	-0.81	4.03	3.17	0.00	6.40
6-09	-0.30	-8.64	2.57	0.00	-6.38
6-10	-0.45	0.66	2.64	0.00	2.86
6-11	-0.37	5.43	2.96	0.00	8.02
6-12	-0.48	-6.29	3.38	0.00	-3.40
6-13	-0.25	3.50	2.58	0.00	5.83
6-14	-0.47	15.00	3.05	0.01	17.60
6-15	-0.79	10.82	3.52	0.02	13.57
6-16	-0.36	-12.38	2.86	0.01	-9.87
x=7					
7-01	0.26	-26.74	0.97	0.00	-25.50
7-02	-0.02	-24.22	1.97	0.00	-22.27
7-03	0.10	-25.29	1.69	0.00	-23.49
7-04	-0.05	-24.46	1.92	0.00	-22.59
7-05	-0.12	-24.31	2.21	0.00	-22.22
7-06	0.00	-16.61	1.88	0.00	-14.74
7-07	-0.06	-15.21	1.80	0.00	-13.47
7-08	0.19	-17.62	1.41	0.00	-16.03

7-09	-0.08	-19.50	1.85	0.00	-17.72
7-10	0.03	-20.99	1.43	0.00	-19.53
7-11	-0.03	-14.19	2.13	0.00	-12.10
7-12	0.08	-20.28	1.76	0.00	-18.44
7-13	0.06	-11.90	1.64	0.00	-10.21
7-14	-0.06	-10.64	2.14	0.00	-8.56
7-15	-0.77	2.88	2.52	0.00	4.63
7-16	-0.31	-5.52	2.08	0.00	-3.76
7-17	0.07	-16.00	1.52	0.00	-14.41
7-18	-0.11	8.25	1.84	0.01	9.99
7-19	-0.19	-13.44	1.64	0.00	-11.98
7-20	-0.25	-23.75	2.36	0.00	-21.65
7-21	-0.07	-25.52	1.90	0.00	-23.68
7-22	-0.14	-15.77	1.98	0.00	-13.93
7-23	-0.09	11.33	1.57	0.04	12.84
7-24	-0.37	6.41	2.14	0.01	8.19
7-25	-0.58	10.13	2.66	0.02	12.23
7-26	-0.08	-20.74	2.24	0.00	-18.57
7-27	-0.37	1.51	2.39	0.00	3.54
7-28	-0.76	11.19	3.32	0.01	13.75
7-29	-0.08	12.20	1.86	0.07	14.05
x=8					
8-01	0.06	-27.60	1.38	0.00	-26.16
8-02	0.03	-28.17	1.56	0.00	-26.58
8-03	0.02	-27.17	1.57	0.00	-25.58
8-04	0.02	-17.72	1.61	0.00	-16.09
8-05	0.24	-20.20	1.08	0.00	-18.88
8-06	0.03	-20.46	1.48	0.00	-18.96
8-07	0.22	-20.89	1.13	0.00	-19.54
8-08	-0.01	-22.72	1.63	0.00	-21.11
8-09	0.17	-29.18	0.51	0.00	-28.50
8-10	-0.28	-10.88	1.94	0.00	-9.22
8-11	0.27	-20.45	1.10	0.00	-19.08
8-12	0.21	-24.34	1.22	0.00	-22.91
8-13	0.30	-22.11	1.53	0.00	-20.28
8-14	-0.06	-13.86	1.82	0.00	-12.10
8-15	0.06	-22.99	1.49	0.00	-21.44
8-16	0.12	-23.11	1.24	0.00	-21.74
8-17	0.08	-24.94	1.40	0.00	-23.47
8-18	-0.35	-4.36	1.95	0.00	-2.76
8-19	0.09	-25.03	1.62	0.00	-23.31
8-20	-0.42	-4.14	1.79	0.00	-2.77
8-21	-0.07	-11.84	1.93	0.00	-9.97
8-22	-0.11	-12.46	1.46	0.00	-11.11

8-23	0.00	-13.74	1.53	0.00	-12.21
8-24	0.01	-18.12	1.43	0.00	-16.67
8-25	-0.25	-7.80	2.04	0.00	-6.00
8-26	-0.61	-0.13	1.89	0.01	1.15
8-27	-0.15	-12.85	2.00	0.00	-11.00
8-28	0.13	-22.94	1.06	0.00	-21.75
8-29	-0.23	-10.18	2.24	0.00	-8.16
8-30	-0.18	-6.49	1.87	0.00	-4.81
8-31	-0.04	-10.15	1.59	0.00	-8.60
8-32	0.06	-6.96	0.82	0.00	-6.08
8-33	-1.07	4.00	3.38	0.01	6.32
8-34	-0.70	4.34	2.53	0.01	6.18
8-35	0.06	-25.20	1.33	0.00	-23.81
8-36	-0.77	10.22	2.97	0.01	12.43
8-37	-0.12	15.96	1.73	0.03	17.60
8-38	-0.76	-2.10	3.23	0.00	0.38

Table S3. The xyz coordinates (in Å) of the lowest-lying isomers of S_x^{2-} and Li_2S_x (x=4-8) clusters.

atom	x	y	z
S_4^{2-}			
S	-2.349431	-0.637591	-0.205166
S	2.349322	-0.637700	0.205093
S	0.885897	0.637711	-0.543996
S	-0.885788	0.637580	0.544069
S_5^{2-}			
S	-3.062781	0.429114	-0.425973
S	1.498433	0.874574	-0.095143
S	3.062972	-0.426452	-0.427922
S	-0.000055	-0.003066	1.048248
S	-1.498569	-0.874169	-0.099210
S_6^{2-}			
S	3.777323	0.440706	-0.124692
S	-0.652587	0.816252	-0.614109
S	-2.183239	0.518967	0.738266
S	0.652438	-0.816542	-0.613824
S	-3.777167	-0.440963	-0.124362
S	2.183232	-0.518420	0.738721
S_7^{2-}			
S	4.421137	0.611071	0.143628
S	0.000077	0.926575	-0.000249
S	-1.354450	-0.292241	0.994027
S	1.354553	-0.293076	-0.993998
S	-2.930170	-0.781530	-0.242269
S	2.930097	-0.781585	0.242810
S	-4.421244	0.610786	-0.143950
S_8^{2-}			
S	-5.059595	-0.681252	-0.334053
S	5.059261	-0.681970	0.333467
S	-0.688586	-0.481848	-0.764156
S	0.688772	-0.482088	0.763995
S	-2.111669	0.974895	-0.345491
S	2.111776	0.975037	0.346109
S	-3.619710	0.188164	0.813681
S	3.619751	0.189061	-0.813551
Li_2S_4			
Li	-0.222175	-1.072155	1.403017
Li	0.222182	-1.072107	-1.403052
S	1.815627	-0.782213	0.256270

S	-0.952147	0.983238	0.427291
S	-1.815627	-0.782213	-0.256269
S	0.952147	0.983237	-0.427285
Li_2S_5			
Li	-0.406907	1.372351	-1.225697
Li	-0.136281	0.936739	1.477453
S	-2.149560	0.934104	0.247970
S	0.185564	-1.422036	0.588439
S	-1.407057	-0.812179	-0.615639
S	1.857687	-0.589734	-0.269441
S	1.615215	1.456891	0.001467
Li_2S_6			
Li	-0.330484	1.384146	1.300897
Li	0.330685	1.384269	-1.299004
S	-0.932744	-1.632997	-0.436240
S	2.060466	-0.221759	-0.547534
S	-2.060686	-0.221454	0.547313
S	0.932512	-1.633009	0.436346
S	1.862706	1.594933	0.430316
S	-1.862291	1.595208	-0.430555
Li_2S_7			
Li	0.000077	1.037500	1.655647
Li	0.001289	1.872162	-0.812652
S	-1.962435	0.284413	-0.994105
S	-0.000277	-1.408420	1.040907
S	1.698368	-1.564694	-0.099030
S	-1.970094	1.712265	0.492283
S	1.971845	1.710444	0.493407
S	1.962561	0.283869	-0.994088
S	-1.700223	-1.563438	-0.097437
Li_2S_8			
Li	-0.764587	1.832543	1.006138
Li	-0.126408	1.103423	-1.488096
S	2.669825	-0.669061	-0.412218
S	-1.372873	-0.816174	1.229583
S	-2.917909	-0.219477	0.026201
S	2.187108	0.687316	1.029280
S	-0.245539	-2.199257	0.179392
S	0.846754	-1.206580	-1.243675
S	-2.314424	1.597495	-0.761687
S	1.314120	2.275246	0.043492
